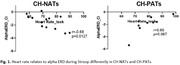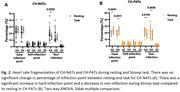# Heart rate fragmentation during a Stroop task reveals cognitively healthy individuals with pathological CSF amyloid/tau

**DOI:** 10.1002/alz.084905

**Published:** 2025-01-09

**Authors:** Elizabeth H. Choy, Abdulhakim Al‐Ezzi, David P Buennagel, Anne Nolty, Alfred N. Fonteh, Robert A Kloner, Michael T. Kleinman, Xianghong Arakaki

**Affiliations:** ^1^ University of California, Irvine, Irvine, CA USA; ^2^ Huntington Medical Research Institutes (HMRI), Pasadena, CA USA; ^3^ Huntington Medical Research Institutes, Pasadena, CA USA; ^4^ Fuller Theological Seminary, Pasadena, CA USA

## Abstract

**Background:**

Stroop task is used to evaluate inhibition, a core executive function. Alpha Event‐Related Desynchronization (ERD) from analysis of electroencephalogram (EEG) during Stroop task reflects brain interference processing. We previously reported different relationships between heart rate variability (HRV) and alpha ERD during Stroop task. However, HRV proxied autonomic function can be confounded by increasing erratic rhythm with age, quantified by heart rate fragmentation (HRF). The knowledge of HRF is limited in a Cognitively Healthy (CH) cohort. To fill this gap, we studied these measurements in CH participants who underwent a Stroop task.

**Method:**

We studied HRF in an established EEG dataset during Stroop task from CH participants when their CSF amyloid/tau ratio were normal (≥2.71, CH‐NATs) or pathological (<2.71, CH‐PATs). Heart rate fragmentation (HRF) was analyzed from 5‐minute electrocardiogram (ECG) during resting or task. HRF was classified into three categories: hard inflection point, soft inflection point, and non‐inflection. Hard inflection point represents an acceleration‐to‐deceleration (AD) in heart rate and vice versa (DA). Soft inflection point describes heart rate transitions from acceleration or deceleration‐to‐zero acceleration (AZ or DZ) and vice versa (ZA or ZD). Non inflection means heart rates are accelerating (NA), decelerating (ND), or no change (NZ).

**Result:**

We previously reported that alpha ERD during incongruent trials were negatively correlated with heart rate (HR) in CH‐NATs but not in CH‐PATs (Figure 1). In CH‐NATs, there was a decrease in a subcategory of non‐inflection during task compared to resting (Figure 2A). We observed a significant increase in hard inflection points and a decrease in non‐inflections during Stroop task compared to resting in CH‐PATs (Figure 2B).

**Conclusion:**

These pilot results suggest: 1) from resting to task, CH‐PATs presented increase of HRF hard inflection points, which was not observed in CH‐NATs; 2) our previous analysis show that HR was negatively correlated with alpha ERD in CH‐NATs (higher HR related to more brain activation ‐ more negative alpha ERD), which was not observed in CH‐PATs. Although a larger sample size is needed, these results support a potential heart‐brain dysregulation using non‐invasive EEG and ECG in cognitively healthy individuals at higher risk of cognitive decline.